# Does AIDS vaccine research offer fresh messages for public health campaigns? A review of public health communication related to HIV AIDS In Uganda

**DOI:** 10.1186/1742-4690-9-S2-P242

**Published:** 2012-09-13

**Authors:** J Nam

**Affiliations:** 1 Olive Branch Media, Kampala, Uganda

## Background

HIV AIDS is a complex scientific phenomenon of our time. Ultimately however, HIV AIDS is a public health matter of unparalleled concern . This is due to the social and economic dimension of the HIV AIDS epidemic in the public domain. Public health communication is an integral part and key instrument employed by national health systems worldwide, in the management of public health.

## Methods

By quantitative and qualitative method of data analysis. This paper investigates t he evolution of public health communication in Uganda related to HIV AIDS in a period covering over two decades. A period that initially placed Uganda as model country in the fight against HIV AIDS globally, as result of the country’s success in decreasing HIV AIDS prevalence rates at national level. It also examines the likely factors behind the recent resurgence in new infections and prevalence rates.

## Results

This paper postulate, giving empirical evidence, that key to the initial success of the fight against HIV AIDS in Uganda were the novelty of public health messages in the face of a fatal disease hitherto unknown by the public, and the means of their delivery. It argues that with the passage of time and reduction in stigma associated with being an HIV AIDS patient, public health messages became over used and ineffective.

## Conclusion

The paper explores possible sources of new public health messages and techniques of dissemination using traditional and new media. It probes whether the on-going research on AIDS Vaccine could generate new messages vital for public health campaigns, without giving a false impression of cure, a tendency that experts concur could lead to the rise in risky behavior in the public in some countries, as has been the case with the introduction of antiretroviral drugs and medical male circumcision.

